# Interventions to Improve Vaccination Uptake and Cost Effectiveness of Vaccination Strategies in Newly Arrived Migrants in the EU/EEA: A Systematic Review

**DOI:** 10.3390/ijerph15102065

**Published:** 2018-09-20

**Authors:** Charles Hui, Jessica Dunn, Rachael Morton, Lukas P. Staub, Anh Tran, Sally Hargreaves, Christina Greenaway, Beverly Ann Biggs, Robin Christensen, Kevin Pottie

**Affiliations:** 1 Division of Infectious Diseases, Children’s Hospital of Eastern Ontario, University of Ottawa, Ottawa, ON K1H 8L1, Canada; jdunn@cheo.on.ca; 2 NHMRC Clinical Trials Centre, Sydney Medical School, University of Sydney, Camperdown 1450, Australia; rachael.morton@ctc.usyd.edu.au (R.M.); lukas.staub@ctc.usyd.edu.au (L.P.S.); anh.tran@ctc.usyd.edu.au (A.T.); 3 International Health Unit, Section of Infectious Diseases and Immunity, Imperial College London; London W12 0NN, UK; s.hargreaves@imperial.ac.uk; 4 The Institute for Infection and Immunity, St George’s, University of London, London SW17 0RE, UK; 5 Division of Infectious Diseases and Clinical Epidemiology, SMBD-Jewish General Hospital, McGill University, Montreal, QC H3T 1E2, Canada; ca.greenaway@mcgill.ca; 6 Department of Medicine/RMH at the Doherty Institute, University of Melbourne, Melbourne 3000, Australia; babiggs@unimelb.edu.au; 7 The Victorian Infectious Diseases Service, Royal Melbourne Hospital, Parkville 3050, Australia; 8 Musculoskeletal Statistics Unit, The Parker Institute, Bispebjerg and Frederiksberg Hospital, 2000 Frederiksberg, Denmark; robin.christensen@regionh.dk; 9 Bruyere Research Institute, Ottawa, ON K1N 5C8, Canada; kpottie@uottawa.ca; 10 Departments of Family Medicine and Epidemiology and Community Medicine, University of Ottawa, Ottawa, ON K1G 5Z3, Canada

**Keywords:** VPD, immunisation strategies, health systems, refugees, migrants, cost effectiveness

## Abstract

Newly arrived migrants to the EU/EEA (arrival within the past five years), as well as other migrant groups in the region, might be under-immunised and lack documentation of previous vaccinations, putting them at increased risk of vaccine-preventable diseases circulating in Europe. We therefore performed a systematic review conforming to PRISMA guidelines (PROSPERO CRD42016045798) to explore: (i) interventions that improve vaccine uptake among migrants; and (ii) cost-effectiveness of vaccination strategies among this population. We searched MEDLINE, Embase, CINAHL, and Cochrane Database of Systematic Reviews (CDSR) between 1 January 2006 to 18 June 2018. We included three primary intervention studies performed in the EU/EEA or high-income countries and one cost effectiveness study relevant to vaccinations in migrants. Intervention studies showed small but promising impact only on vaccine uptake with social mobilization/community outreach, planned vaccination programs and education campaigns. Targeting migrants for catch-up vaccination is cost effective for presumptive vaccination for diphtheria, tetanus, and polio, and there was no evidence of benefit of carrying out pre-vaccination serological testing. The cost-effectiveness is sensitive to the seroprevalence and adherence to vaccinations of the migrant. We conclude that scarce but direct EU/EEA data suggest social mobilization, vaccine programs, and education campaigns are promising strategies for migrants, but more research is needed. Research should also study cost effectiveness of strategies. Vaccination of migrants should continue to be a public heath priority in EU/EEA.

## 1. Introduction

Globally, there are over 258 million people who migrate across international borders, including labour migrants, students, refugees, asylum seekers, undocumented migrants, and other migrant groups [1]. Another 763 million people migrate internally [2]. In the European Union/European Economic Area (EU/EEA) in particular, there has been an unprecedented number of refugees and other migrants between 2014 and 2016 linked to the Syrian war [3], other armed conflicts, climate change and economic crises. In addition, there are around 40 million EU migrants who have moved internally from one European country to another [4].

Control of vaccine preventable diseases (VPDs) is a priority in the EU/EEA [5]. Very little information is available on the occurrence of vaccine preventable diseases specifically among newly arrived migrant populations in the EU/EEA. Although national surveillance systems for VPDs are in place and regular reporting occurs, surveillance is incomplete for migrant health data such as country of birth and time since arrival. There have been EU/EEA measles and polio outbreaks that have been related to under-immunised migrant populations [6,7,8,9,10], but outbreaks have also occurred in non-migrant populations [11,12,13,14]. The 2017/2018 pan-European measles epidemic involved internal EU/EEA migrants moving between countries, so it is important also to consider this group alongside migrants arriving from outside of the EU/EEA [15].

Seroprevalence studies have demonstrated sub-optimal immunity to VPD among adult and child migrants [16,17,18,19,20,21,22,23,24]. Further, the WHO’s global data on immunisation coverage report sub-optimal immunisation among the general population worldwide, with global coverage ranging 47–85% depending on the vaccine and even greater variation between geographical region [25]. This includes the EU/EEA, where some countries have not achieved target vaccine coverage with regards to, for example, first dose measles. Among all types of migrants from the top ten countries of birth arriving to the EU/EEA, the range of age-appropriate (i.e., two-dose) measles vaccination coverage ranges 31–99% [26]. Sub-optimal immunity has implications for maintaining herd immunity to minimize outbreaks where seropositivity thresholds within 80–94% are required [25,27]. Collective immunity below these thresholds, whether it is the native-born population, newly-arrived migrants, or a combination, carries the inherent risk of disease transmission and outbreak.

A recent cross-sectional survey study of EU/EEA countries “immigrant” measles vaccination policy demonstrated a significant diversity in strategies with 9 of 31 states having no policy. The remaining 22 states′ policies differed widely in utilizing age, immunisation status, method for assessing immunisation status and immigrant type [28]. Furthermore, vaccination policies tailored to migrants and refugees are heterogenous across WHO European region member states [29]. Vaccinations are effective, but there are no specific data on effective implementation strategies for immunization in migrants to EU/EEA. We therefore performed a systematic review to address: (i) what interventions increase uptake of vaccinations in migrants; and (ii) cost-effectiveness of vaccination strategies among migrants.

## 2. Methods

Using the Grading of Recommendations Assessment, Development and Evaluation (GRADE) approach, the Campbell and Cochrane Collaboration Equity Methods Group and review team, including clinicians, public health experts and researchers from across the EU/EEA, conducted evidence syntheses. A detailed description of the methods can be found in the registered systematic review protocol [30].

The review group followed the PRISMA Reporting guideline [31] for the reporting of this systematic review (PROSPERO CRD42016045798). In summary, the review team developed key research questions and a logic model showing an evidence chain to identify key concepts, to consider the potential role of indirect evidence related to populations and interventions and to support the formulation of search strategies (Supplementary Materials). We aimed to answer the following questions:

*What interventions increase the uptake of vaccinations in migrants?*

*What are cost-effective strategies for vaccinating migrants?*

We developed our inclusion and exclusion criteria based on the PICO model (population, intervention, comparator and outcome). “Migrants,” a focus for the eligible evidence, included asylum seekers, refugees, undocumented migrants, and other foreign-born residents, with a focus on newly arrived migrants as defined in the protocol as within five years of arrival to the destination country [30]. Internal EU migrants were also included to reflect the large movement of migrants within the EU who were new to the EU/EEA. Only papers addressing migrants to the EU/EEA or other high-income countries were included in the final synthesis. The intervention included any strategy to increase vaccination. The comparators were migrants not exposed to the intervention. We considered the following outcomes: uptake of vaccination and completion of vaccination; disease incidence rates for measles, congenital rubella, diphtheria pertussis, tetanus, Hib, and polio among migrant populations; and cost effectiveness.

Using relevant search terms and strategies, we searched published literature from 1 January 2006 to 18 June 2018 in MEDLINE, Embase, CINAHL, and Cochrane Database of Systematic Reviews (CDSR) for interventions to improve vaccine uptake (File Supplementary A). Searches were designed and conducted by librarian experienced in systematic reviews using a method designed to optimize term selection [32]. The MEDLINE search was validated by testing its ability to retrieve the eligible studies found from the initial search. All were indexed in MEDLINE and the search retrieval was 95%, thus the search was not modified before the final update was run [33]. For economic studies, we searched MEDLINE, Database of Abstracts of Reviews of Effects (DARE), (CDSR) and EMBASE from 1 January 2006 to 26 May 2016, and in addition, NHS EED, CEA Registry (Tufts University) and Google Scholar from 1995 to 2016 (File Supplementary B). No language restrictions were applied to both searches.

For the intervention studies, at least two reviewers (JD, CH, JB, and NN) independently reviewed the titles and abstracts of the papers identified from the search, identifying them as “included” or “not included” based on previously described inclusion/exclusion criteria. Any conflicts were then resolved through discussion by the two review leads (CH and JD). At the second level screening, two independent reviewers performed a full text review per paper (JD, CH, JB, and NN). Conflicts were again resolved via discussion by review leads (CH and JD). The quality of nonrandomized studies was assessed using the Newcastle–Ottawa Scale (NOS) [34]. No studies were excluded based on the NOS.

For the cost effectiveness studies, two reviewers independently reviewed all full text articles (LS and AT) and extracted relevant data from the primary studies that met our inclusion criteria including the economic study design (e.g., micro-costing study, within-trial cost-utility analysis, and decision-analytic model); the intervention and comparator; the difference in resource use; cost-effectiveness results (e.g., incremental net benefit or incremental cost-effectiveness ratio); the certainty of evidence around resource requirements; and whether the cost-effectiveness results favoured the intervention or comparator. Finally, we assessed the certainty of economic evidence in each study using the relevant items from the 1997 Drummond checklist [35].

## 3. Results

### 3.1. What Interventions Increase Uptake of Vaccinations in Migrants?

**Figure 1:**
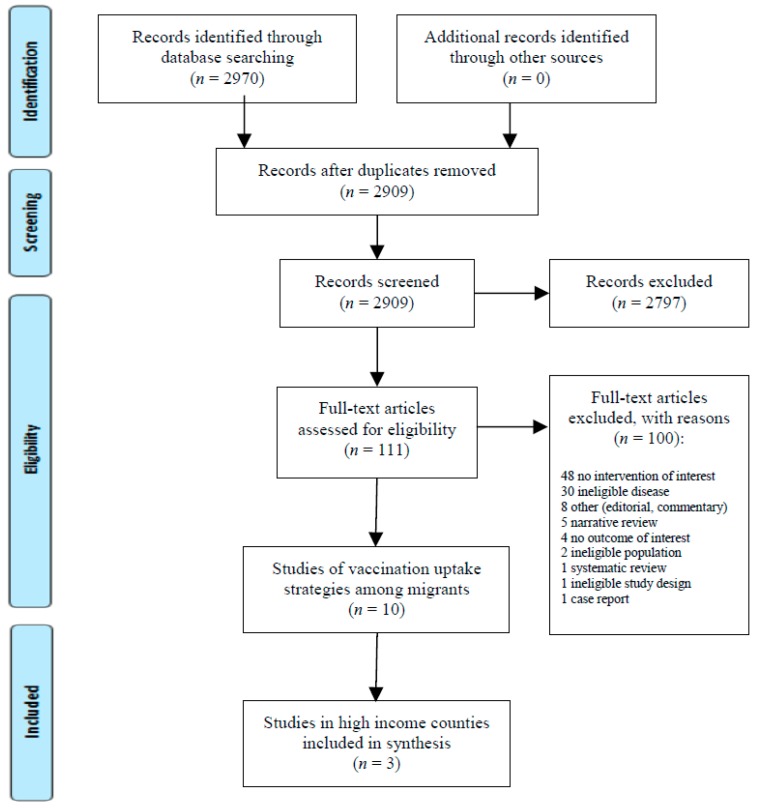
PRISMA Flow Diagram–Interventions to Increase Vaccination Uptake among Migrants.

The search for interventions that increase uptake of vaccinations identified 2970 studies. Three studies were included in the final analysis [36,37,38] (PRISMA Flow Diagram Figure 1). After assessment with the Newcastle–Ottawa Scale (NOS; Table 1), two were determined to be of medium quality [36,38] (NOS 4–6), and one was a low quality study [37] (NOS < 4).

Two studies were in international migrants to Germany [38] and Australia [36] and one was in internal migrants in Italy [37]. The study characteristics are described in Table 1. Interventions included social mobilization/community outreach [38], planned vaccination programs [36,37], and education campaigns [38]. All the published interventions were associated with positive outcomes of receipt of vaccination. The only outcome reported was vaccine uptake. No disease rates, enrolment in health services or migrant acceptance of vaccination were reported.

Of the two studies that were performed in Europe, one was cohort study of asylum seekers living in housing units in Germany employed a vaccination strategy using multiple different interventions [38]. The local public health office informed asylees about relevant VPDs in written letters and posters, as well as in person, and invited them to on-site vaccination campaigns. General practitioners carried out the actual vaccination. Information material regarding vaccination was provided in various languages and via interpreters. Vaccination “certificates” were also provided. In areas utilizing this vaccination strategy, 58% of refugees were vaccinated compared to 6% of refugees vaccinated in facilities without the intervention. Of the total 642 asylees who were vaccinated, 86% received their immunization within the vaccine intervention program. There was a particular focus on male adult asylees who had an eight-fold increase in vaccinations through the strategy. Of note, the program purchased vaccines directly from the manufacturer, saving 50% of the cost compared to buying from a pharmacy. A second European study involved Roma children and women of childbearing age in a nomadic camp in Rome. As part of a tuberculosis outbreak assessment, a monthly vaccination day led to a 56% coverage of hexavalent vaccines and a 58% coverage of MMR vaccines which was a 30% increase in vaccinated subjects compared with the previous year [37]. The third study that was performed in a high income country was a study of refugee adolescents and young adults in an Intensive English Centre high school in Australia employed a survey of immunisation status [36]. The intervention involved the school-based provision of MMR and the first and second dose of a three-dose hepatitis B schedule following an immunisation survey. Of the 165 students who completed the survey (85%), 74% received measles, mumps and rubella (MMR) vaccine in the school as compared with historic levels of 30%. Of the students who received a second dose of hepatitis B vaccine in the school-based program, less than 24% finished the series with a primary care clinician.

### 3.2. What are Cost-Effective Approaches to Vaccinating Newly Arrived Migrants?

**Figure 2:**
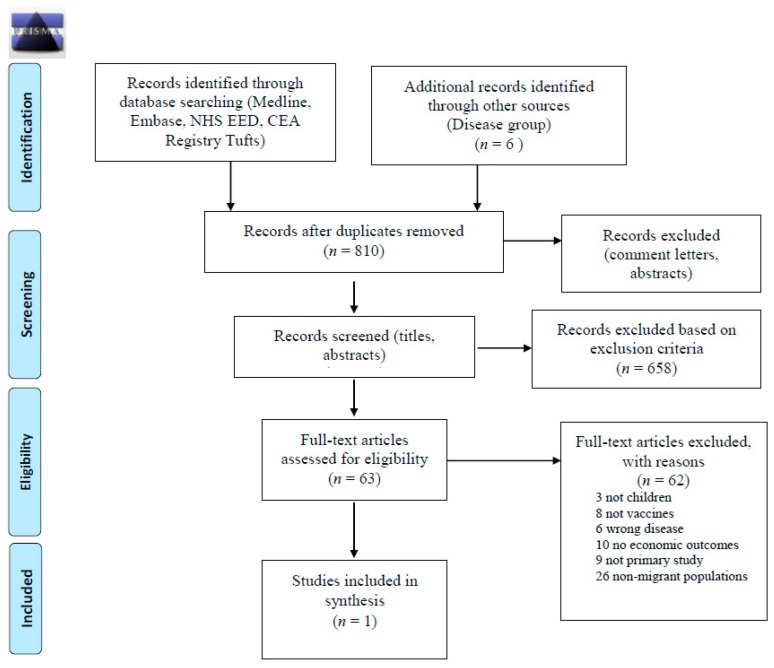
PRISMA 2009 Flow Diagram for Cost-Effectiveness of Vaccination Strategies.

We identified 810 articles for screening. One article conducted among migrants was included in the final analysis [39] (PRISMA flow diagram Figure 2). This cost-effectiveness study was assessed to be of moderate quality using the Drummond checklist. One-way and two-way sensitivity analyses were undertaken, and the cost-effectiveness results were tested for changes across plausible ranges of estimates for costs of serotesting, compliance rates and seroprevalence. Probabilistic sensitivity analyses were not reported.

Results of the economic study are presented in Table 2. This study among migrants compared pre-vaccination serotesting with presumptive vaccination for polio, diphtheria, and tetanus in internationally adopted and immigrant infants to the US [39]. It showed that compared with presumptive vaccination, pre-vaccination serotesting for polio increased the cost per patient from $57 USD to $62 USD (in 2004 dollars) and decreased the percentage of patients protected against polio from 95.3% to 94.0%. Presumptive vaccination was more effective and less expensive than pre-vaccination serotesting when the seroprevalence was <69%. Presumptive vaccination was the preferred method unless the vaccination compliance was extremely high (>96% completion rate). In the same study, the results for diphtheria, tetanus, and acellular pertussis (DTaP) were less definitive. Pre-vaccination serotesting for diphtheria and tetanus increased the cost per patient from $62 USD to $119 USD and increased the percentage of patients protected against both diphtheria and tetanus from 91.5% to 92.3%. Presumptive vaccination was the preferred strategy (with an incremental cost-effectiveness ratio (ICER) of $7148 USD per infant protected) in populations with poor vaccine compliance (where >80% of patients did not complete the full catch-up vaccine series), or populations with low seroprevalence (<51%) of antibodies to diphtheria and tetanus.

## 4. Discussion

This systematic review identified few data on interventions to increase vaccinations and cost-effectiveness of vaccinations in migrant populations. Several interventions were identified as potentially helpful to increase vaccination rates in migrant populations. The interventions focused on social mobilization and outreach programs, planned vaccinations, and educational campaigns similar to strategies used to improve vaccination rates in low to middle income countries in migrant populations [40,41,42,43,44]. Overall, the interventions in this study described did not address all the vaccination barriers in migrants such as: use of traditional health care [45], socioeconomic status [45], language [46], distance to vaccination service [46,47], continued migration [47], fear of arrest [47], necessity of work [47], lack of vaccination knowledge [46,48,49], cost [49], and lack of health care provider recommendation [50]. None of the interventions focused on health care providers. The interventions were not targeted, having the same strategy for different migrant age groups and all of the interventions were focused on groups of migrants, not at the level of the individual patient-healthcare interaction. Additionally, none of the studies identified that they had engaged migrant populations in the planning or execution of the intervention.

Vaccinations should not be provided in isolation, but the interaction should be viewed as an opportunity to address many important diseases of public health significance [51]. Engaging refugees and other migrant populations in preventive health services remains a challenge in light of the barriers to healthcare [52,53]. A recent consensus statement on access to health services in the EU/EEA by IOM’s EQUI HEALTH project [54] highlights the discrepancies in entitlements to statutory health services for migrants; irregular migrants often have highly restrictive access.

The number of studies on the economic analysis for vaccinations in migrant populations is even more limited. Only one study was applicable, suggesting that presumptive polio and DTaP vaccination appear to be more cost effective and less expensive than pre-vaccination serotesting [39]. This modelling was done utilizing data from a very small and unique population of international migrants, international adoptees. Although the economic analysis demonstrated cost effectiveness of vaccination without serological testing, this group has significant resources and understanding of the health systems and entitlements of the country of destination and engages with the health care system on an individual basis. The cost effectiveness differed depending on the serological prevalence of the VPDs and the compliance with vaccinations. Seroprevalence can vary depending on country of origin, type of migrant and age of migrant making it difficult to extrapolate these data.

Two studies published after the economic analysis systematic review was performed examined different costs associated with pre-departure vaccinations, one in the context of a response to an outbreak [55] and the second evaluating the US Vaccination Program for US-bound Refugees (VPR) [56]. The first study showed that pre-departure vaccination of all US bound refugees would not only improve health, reduce importations of VPD, but be cost saving when considering all the resources required for response to outbreak activities. The second US study demonstrated that compared with post-arrival vaccinations, the initiation of the pre-departure VPR where the refugees received one or two doses of selected vaccines before departure and completed the series after arrival demonstrated a net savings per person of $225.93 USD (29% decrease in vaccination costs). The cost savings were sensitive to different variables but demonstrated cost savings across all the estimates. Although the European context is not the same as the orderly US Refugee program, these data could be used to support the vaccination in reception centres before the onward migration in the EU/EEA. Not only is it cost-saving, but there is the potential to prevent unwanted and costly outbreaks of VPDs.

## 5. Strengths and Limitations

Our study is the first to our knowledge that is a systematic review on interventions that increase vaccinations in migrant populations and cost effectiveness of vaccination strategies in migrant populations. The studies that we found reflect the overall migration health literature of low quality with many being cohort or pre-post studies and no negative studies or long term follow up studies. Other limitations of our study are the lack of comparator data and the fact that the only reported outcome was vaccine uptake. Given the complexities of immunisations in this vulnerable population, a short-term increase in vaccine uptake does not directly translate into decreased VPD incidence or decrease in outbreaks. Finally, there is difficulty in extrapolating this evidence across the very heterogeneous group of migrants (undocumented migrant, refugee, asylum seeker, labour migrant, unaccompanied children, etc.) with different pre-migration immunisation coverage, immunity, and age.

## 6. Implementation Considerations and Evidence Gaps

The lack of data on migrant specific variables (i.e., country of birth) related to VPDs [7,57,58,59] makes estimation of the scope of vaccine delivery challenging. Small studies done in special circumstances have shown some sub-optimal vaccine coverage and immunity, but there is a limit to how these data can be extrapolated to all migrants [16,18,19,20,22,23,24]. Robust surveillance data on vaccine preventable diseases and vaccine coverage in migrant populations by age group, migration type, source country, and duration of presence in the EU/EEA will be required to design effective immunisation programmes [57,58]. This will require a standardisation of migrant definitions and parameters. Further research on vaccination uptake, immunisation coverage, and cost-effectiveness of different strategies in adults versus children is required to inform potential different migrant guidelines [60]. The optimal method to document immunisations and share immunisation data across jurisdictions in mobile populations to ensure that the migrant receives the correct immunisations at the appropriate time is an understudied area [61]. Documentation could be done via a standardised health record or mobile immunisation record [62,63]. However, they need to be secure, private, and ensure that the information does not disadvantage or be reason for persecution of the migrant. Engaging migrants in the development of interventions is important to the development of effective interventions. Finally, evidence on the comparative effective implementation strategies and cost-effectiveness of different vaccination strategies for migrants will be required to prioritize the VPDs prevention efforts for the EU/EEA.

## 7. Conclusions

High quality studies assessing interventions to increase vaccinations and the cost effectiveness of vaccinations in migrant populations are scarce. Data on migrant populations, vaccine preventable diseases and vaccinations are required to estimate the scale of the problem and to understand the benefit of interventions on a population scale. Large scale studies on interventions to improve vaccination uptake among different typologies of migrants, and across their migration journey, are required to inform the best and most equitable care of migrants. The economic analyses of these interventions are crucial to inform their implementation.

## Figures and Tables

**Table 1:** Characteristics of included studies in high income countries—strategies to increase vaccination uptake.

Study	Quality ^1^	Type of study	Setting (Country)	Population	Intervention	Results/Outcomes
Brockmann, 2016 [38]	4/10	Cohort study	Housing units (Germany)	Children, adolescent, adult asylum seekers	Vaccination “concept” facilitated by local public health office: (1) Written letters and posters informing about VPDs (2) In person communication about VPDs (3) Invitations to onsite vaccination campaigns (4) Informational vaccine material in various languages and via interpreters	58% of refugees exposed to concept were vaccinated compared to 6% of refugees vaccinated in facilities without the intervention
Milne, 2006 [36]	4/10	Cross-sectional: assessing uptake of MMR, HepB	School (Australia)	Refugee adolescents, young adults	(1) Self-report survey on immunisation status and primary health care use, with provision of 1 dose MMR. (2) Letter given to student with due date f and written referral to GP; list of GPs and spoken languages available	74% students received MMR vaccine 30% historical vaccination rate
Spadea, 2014 [37]	2/10	Cross-sectional: assessing uptake of MMR and hexavalent (DPT-Hib-IPV-HepB)	Nomadic camp (Italy)	Roma children and women of childbearing age	Vaccination day held on monthly basis	56.4% coverage of hexavalent vaccine (range 44–91%) at three camps 58.4% coverage of MMR vaccine (range 53–83%) at three camps 30% increase in vaccinations compared with previous year

^1^ The quality of evidence was assessed using the Newcastle–Ottawa Scale (NOS); rated out of 10 for cross-sectional studies, and out of 9 for case-control or cohort studies.

**Table 2:** Characteristics of Studies—Cost-Effectiveness of Vaccination Strategies.

Study	Certainty of Economic Evidence (Quality)	Design	Population	Intervention	Cost-Effectiveness	Resource Requirements
Cohen et al. 2006 [39]	Some allowance made for uncertainty in the estimates of costs and consequences. The costs are provided as base case, and 25% upper and lower range No probabilistic sensitivity analyses performed. Sensitivity analysis was undertaken for costs of serotesting, compliance rate and seroprevalence. Cost-effectiveness results were sensitive to changes in seroprevalence, cost of serotesting.	Decision-analytic model; results presented in 2004 US dollars	US	1. presumptive vaccination with IPV 2. serotesting for poliovirus type 1, 2, and 3 antibodies followed by vaccination in unprotected patients	For IPV, presumptive vaccination is less costly and more effective, For Dtap, ICER is $7148 USD per person protected.	Difference in costs between 2 interventions are small. For IPV, difference in cost is very minimal: Serotesting is slightly more expensive ($5 USD) than presumptive vaccination. For Dtap, serotesting is more expensive than presumptive vaccine ($57 USD)

## Supplementary Materials

The following are available online at http://www.mdpi.com/1660-4601/15/10/2065/s1. Table S1. PRISMA 2009 Checklist. File A: Electronic search strategies—interventions to increase vaccination strategies. File B: Electronic search strategies—economic studies.
