# Supplementary material for: Interventions to Improve Vaccination Uptake and Cost Effectiveness of Vaccination Strategies in Newly Arrived Migrants in the EU/EEA: A Systematic Review
Source: Int J Environ Res Public Health. 2018 Sep 20;15(10):2065. doi: 10.3390/ijerph15102065 (PMC6210200; doi:10.3390/ijerph15102065)
Supplement: Supplementary file 1 [file ijerph-15-02065-s001.pdf]

Supplementary materials

Supplementary Table S1. PRISMA 2009 Checklist.

Section/topic	#	Checklist item	Reported on page #
		TITLE	
Title	1	Identify the report as a systematic review, meta-analysis, or both.	1
		ABSTRACT	
Structured summary	2	Provide a structured summary including, as applicable: background; objectives; data sources; study eligibility criteria, participants, and interventions; study appraisal and synthesis methods; results; limitations; conclusions and implications of key findings; systematic review registration number.	1
		INTRODUCTION	
Rationale	3	Describe the rationale for the review in the context of what is already known.	2
Objectives	4	Provide an explicit statement of questions being addressed with reference to participants, interventions, comparisons, outcomes, and study design (PICOS).	2
		METHODS	
Protocol and registration	5	Indicate if a review protocol exists, if and where it can be accessed (e.g., Web address), and, if available, provide registration information including registration number.	2
Eligibility criteria	6	Specify study characteristics (e.g., PICOS, length of follow-up) and report characteristics (e.g., years considered, language, publication status) used as criteria for eligibility, giving rationale.	3
Information sources	7	Describe all information sources (e.g., databases with dates of coverage, contact with study authors to identify additional studies) in the search and date last searched.	3
Search	8	Present full electronic search strategy for at least one database, including any limits used, such that it could be repeated.	3
Study selection	9	State the process for selecting studies (i.e., screening, eligibility, included in systematic review, and, if applicable, included in the meta-analysis).	3
Data collection process	10	Describe method of data extraction from reports (e.g., piloted forms, independently, in duplicate) and any processes for obtaining and confirming data from investigators.	3
Data items	11	List and define all variables for which data were sought (e.g., PICOS, funding sources) and any assumptions and simplifications made.	3
Risk of bias in individual studies	12	Describe methods used for assessing risk of bias of individual studies (including specification of whether this was done at the study or outcome level), and how this information is to be used in any data synthesis.	3
Summary measures	13	State the principal summary measures (e.g., risk ratio, difference in means).	n/a
Synthesis of results	14	Describe the methods of handling data and combining results of studies, if done, including measures of consistency (e.g., I^2) for each meta-analysis.	n/a

Supplementary A) Electronic search strategies – Interventions to increase vaccination strategies

Note: Searches were conducted using an Ovid multi-database search. Lines 1–8 are optimized for Embase and the main question constructs are broken out in separate lines for clarity. Lines 9–12 are optimized for MEDLINE and lines 3–17 are optimized for CENTRAL. The next lines isolate the records to the database the search was designed for, combine those sets and then remove duplicate records and final isolate the records from each database again so each can be downloaded and imported into the citation manager using a database-specific import filter.

1. exp "Transients and Migrants"/ or exp "Emigrants and Immigrants"/ or exp Refugees/ or exp Homeless Persons/ or Medically Uninsured/ or (migrant* or migrat* or immigrant* or refugee* or displaced person* or transients or (transient adj3 (peop* or pers* or pop* or worker*)) or nomad* or asylum seek* or (migratory adj3 (peop* or pers* or pop* or worker*)) or alien* or emigrant* or expatriate* or Uninsured or , homeless* or street pe* or underserv* or under serv* or uninsure*).ti,ab,kf.
2. exp Vaccination/ or exp Treatment Refusal/ or Measles-Mumps-Rubella Vaccine/ or exp Vaccines/ad, og, sd, ut or ((vaccin* or immuniz* or immunis* or innoculat* or MMR or measles mumps or unvaccin* or unimmuniz* or unimmunis*) adj3 (rate* or target* or uptake or service* or delivery or system* or strateg* or program* or campaign* or accept* or barrier* or resist* or refus* or concern* or reluct* or renounc* or willing* or unwilling* or cooperat*)).ti,ab,kf.
3. (1 and 2) not (animal/ not human/)
4. exp Migrant/ or Homelessness/ or Medically Uninsured/ or (migrant* or migrat* or immigrant* or refugee* or displaced person* or transients or (transient adj3 (peop* or pers* or pop* or worker*)) or nomad* or asylum seek* or (migratory adj3 (peop* or pers* or pop* or worker*)) or alien* or emigrant* or expatriate* or Uninsured or homeless* or street pe* or underserv* or under serv* or uninsure*).ti,ab,kw.
5. exp Vaccination/ or exp Treatment Refusal/ or exp Immunisation/ or exp vaccine/ad, dt or ((vaccin* or immuniz* or immunis* or innoculat* or MMR or measles mumps or unvaccin* or unimmuniz* or unimmunis*) adj3 (rate* or target* or uptake or service* or delivery or system* or strateg* or program* or campaign* or accept* or barrier* or resist* or refus* or concern* or reluct* or renounc* or willing* or unwilling* or cooperat*)).ti,ab,kw.
6. 4 and 5
7. limit 6 to humans
8. limit 6 to animals
9. 6 not (8 not 7)
10. (migrant* or migrat* or immigrant* or refugee* or displaced person* or transients or (transient adj3 (peop* or pers* or pop* or worker*)) or nomad* or asylum seek* or (migratory adj3 (peop* or pers* or pop* or worker*)) or alien* or emigrant* or expatriate* or Uninsured or homeless* or street pe* or underserv* or under serv* or uninsure*).ti,ab,kw.
11. ((vaccin* or immuniz* or immunis* or innoculat* or MMR or measles mumps or unvaccin* or unimmuniz* or unimmunis*) adj3 (rate* or target* or uptake or service* or delivery or system* or strateg* or program* or campaign* or accept* or barrier* or resist* or refus* or concern* or reluct* or renounc* or willing* or unwilling* or cooperat*)).ti,ab,kw.
12. 10 and 11
13. 3 use ppez
14. 9 use emez
15. limit 14 to embase
16. 12 use cctr
17. 13 or 15 or 16
18. limit 17 to yr="2006 - 2017"
19. remove duplicates from 18

20. 19 use ppez
21. 19 use emez
22. 19 use cctr

CINAHL
S1 (MH "Transients and Migrants+" or MH "Emigrants and Immigrants+" or MH Refugees+ or MH Homeless Persons+ or Medically Uninsured+ or (migrant* or migrat* or immigrant* or refugee* or displaced person* or transients or transient p* or nomad* or asylum seek* or migratory p* or alien* or emigrant* or expatriate* or Uninsured or , homeless* or street pe* or underserv* or under serv* or uninsure*)) AND (MH Immunisation+ or MH Treatment Refusal+ or MH "Vaccines+/SD/AD" or ((vaccin* or immuni* or unvaccin* or unimmuni*) N2 (rate* or target* or uptake or service* or delivery or system* or strateg* or program* or campaign* or accept* or barrier* or resist* or refus* or concern* or reluct* or renounc* or willing* or unwilling* or cooperat*)))
Limiters - Published Date: 20060101–20171231; Exclude MEDLINE records

Supplementary B) Electronic search strategies – economic studies

Database: Ovid MEDLINE(R) Epub Ahead of Print <May Week 3 2016>, Ovid MEDLINE(R) 1946 to Present with Daily Update
Search Date: 27 May 2016
----
1 exp Measles/ (14148)
2 exp Measles virus/ (6086)
3 measles.tw. (19365)
4 (rubeola or rubeolla).tw. (303)
5 Mumps/ (4188)
6 Mumps virus/ (1957)
7 mumps.tw. (6794)
8 ((epidemic or infectious) adj parotiti$).tw. (376)
9 exp Rubella/ (7731)
10 Rubella virus/ (3319)
11 rubella?.tw. (10698)
12 german measles.tw. (205)
13 mmr.mp. (5350)
14 exp Poliomyelitis/ (19115)
15 Poliovirus/ (9384)
16 polio$.mp. (32314)
17 Tetanus/ (9148)
18 tetanus.tw. (20382)
19 Diphtheria/ (6428)
20 Diphtheria toxin/ (3358)
21 diphtheria?.tw. (13334)
22 (dtp or dtap or tdap).tw. (2066)
23 Whooping Cough/ (7494)
24 whooping cough.tw. (2931)
25 Bordetella pertussis/ (4779)
26 pertuss$.tw. (23704)
27 Meningitis, Haemophilus/ (2406)
28 Haemophilus influenzae/ (11946)
29 Haemophilus influenzae type b/ (1044)
30 ((haemophilus or hemophilus) adj influenzae).tw. (16317)

31 "h. influenzae".tw. (5959)
32 hib.tw. (2608)
33 or/1-32 (143642)
34 exp Vaccination/ (70944)
35 exp Immunisation/ (153306)
36 (immunis$ or immuniz$ or vaccinat$ or vaccine?).mp. (369301)
37 or/34-36 (380206)
38 meta analysis.mp,pt. (96691)
39 review.pt. (2060242)
40 search$.tw. (266622)
41 guideline.pt. (15762)
42 guideline/ (15762)
43 guidelines as topic/ (34055)
44 practice guideline.pt. (21205)
45 practice guideline/ (21205)
46 practice guidelines as topic/ (91766)
47 (CPG or CPGs or guidance or guideline? or recommend$ or standard?).ti. (147108)
48 exp clinical pathway/ (5271)
49 exp clinical protocol/ (139305)
50 ((care or clinical) adj2 pathway?).tw. (5123)
51 or/38-50 (2571208)
52 33 and 37 and 51 (5144)
53 animals/ not (humans/ and animals/) (4214645)
54 52 not 53 (5025)
55 54 and (2006$ or 2007$ or 2008$ or 2009$ or 2010$ or 2011$ or 2012$ or 2013$ or 2014$ or 2015$ or 2016$).ed. (2004)
56 remove duplicates from 55 [reviews and guidelines] (1957)
57 exp "costs and cost analysis"/ (197876)
58 cost$.mp. (467657)
59 cost effective$.tw. (83040)
60 cost benefit analys$.mp. (67297)
61 health care costs.mp. (37140)
62 or/57-61 (476991)
63 33 and 37 and 62 (1677)
64 animals/ not (humans/ and animals/) (4214645)
65 63 not 64 (1597)
66 65 and (2006$ or 2007$ or 2008$ or 2009$ or 2010$ or 2011$ or 2012$ or 2013$ or 2014$ or 2015$ or 2016$).ed. (618)
67 remove duplicates from 66 [costing] (605)
****

Database: Embase <1974 to 2016 May 26>
Search Date: 27 May 2016
----
1 measles/ (17908)
2 measles virus/ (8373)
3 measles.tw. (22265)
4 (rubeola or rubeolla).tw. (375)
5 mumps/ (7025)
6 mumps virus/ (2383)
7 mumps.tw. (8103)
8 ((epidemic or infectious) adj parotiti$).tw. (395)

9 rubella/ (10608)
10 rubella virus/ (4531)
11 rubella?.tw. (12837)
12 german measles.tw. (263)
13 mmr.mp. (9200)
14 poliomyelitis/ (21668)
15 exp poliomyelitis virus/ (9757)
16 polio$.mp. (39806)
17 tetanus/ (14449)
18 tetanus.tw. (23054)
19 diphtheria/ (10727)
20 diphtheria toxin/ (4608)
21 diphtheria?.tw. (15084)
22 (dtp or dtap or tdap).tw. (2833)
23 pertussis/ (12134)
24 pertuss$.tw. (26921)
25 whooping cough.tw. (3250)
26 haemophilus meningitis/ (379)
27 exp haemophilus influenzae/ (26673)
28 ((haemophilus or hemophilus) adj influenzae).tw. (19114)
29 "h. influenzae".tw. (7290)
30 hib.tw. (3335)
31 or/1-30 (179278)
32 exp vaccination/ (136796)
33 exp immunisation/ (240553)
34 (immunis$ or immuniz$ or vaccinat$ or vaccine?).mp. (478498)
35 or/32-34 (484710)
36 meta analys$.mp. (170912)
37 search$.tw. (371891)
38 review.pt. (2163167)
39 guideline.pt. (0)
40 guideline/ (144)
41 guidelines as topic/ (229891)
42 practice guideline.pt. (0)
43 practice guideline/ (275498)
44 practice guidelines as topic/ (171087)
45 (CPG or CPGs or guidance or guideline? or recommend$ or standard?).ti. (203281)
46 exp clinical pathway/ (6983)
47 exp clinical protocol/ (75932)
48 ((care or clinical) adj2 pathway?).tw. (9455)
49 or/36-48 (2900815)
50 31 and 35 and 49 (9135)
51 (exp animal/ or animal.hw. or nonhuman/) not (exp human/ or human cell/ or (human or humans).ti.) (5865316)
52 50 not 51 (8806)
53 (immigra$ or migrant$ or migration$ or refugee$).mp. (337937)
54 52 and 53 and (2006$ or 2007$ or 2008$ or 2009$ or 2010$ or 2011$ or 2012$ or 2013$ or 2014$ or 2015$ or 2016$).dd. (93)
55 remove duplicates from 54 [reviews and guidelines] (93)
56 cost effectiveness analysis/ (114261)
57 cost.tw. (387424)
58 costs.tw. (208729)

59 or/56-58 (544762)
60 31 and 35 and 59 (2408)
61 (exp animal/ or animal.hw. or nonhuman/) not (exp human/ or human cell/ or (human or humans).ti.) (5865316)
62 60 not 61 (2304)
63 (immigra$ or migrant$ or migration$ or refugee$).mp. (337937)
64 62 and 63 and (2006$ or 2007$ or 2008$ or 2009$ or 2010$ or 2011$ or 2012$ or 2013$ or 2014$ or 2015$ or 2016$).dd. (19)
65 remove duplicates from 64 [costing] (19)
****

Database: EBSCO CINAHL <1970 to April 2016>
Search Date: 27 April 2016

Query Limiters/Expanders Last Run Via Results
S38 S32 AND S37 Limiters - Exclude MEDLINE records 155
S37 S14 AND S17 AND S36 684
S36 S34 OR S35 139,642
S35 TI (cost OR costs) OR AB (cost OR costs) 89,494
S34 (MH "Costs and Cost Analysis+") 82,899
S33 S29 AND S32 Limiters - Exclude MEDLINE records 498
S32 S30 OR S31 3,831,894
S31 EM 2006 OR EM 2007 OR EM 2008 OR EM 2009 OR EM 2010 OR EM 2011 OR EM 2012 OR EM 2013 OR EM 2014 OR EM 2015 OR EM 2016 3,625,704
S30 PY 2006 OR PY 2007 OR PY 2008 OR PY 2009 OR PY 2010 OR PY 2011 OR PY 2012 OR PY 2013 OR PY 2014 OR PY 2015 OR PY 2016 3,473,513
S29 S14 AND S17 AND S28 1,882
S28 S18 OR S19 OR S20 OR S21 OR S22 OR S23 OR S24 OR S25 OR S26 OR S27 348,095
S27 TX (care or clinical) N2 pathway* 15,546
S26 TI (CPG or CPGs or guidance or guideline* or recommend* or standard*) 79,217
S25 (MH "Critical Path") 4,120
S24 PT Practice Guidelines 9,486
S23 (MH "Practice Guidelines") 53,686
S22 (TI meta analy* or AB meta analy*) 30,459
S21 (MH "Meta Analysis") 25,196
S20 PT review 143,877
S19 PT systematic review 53,350
S18 (MH "Systematic Review") 37,837
S17 S15 OR S16 98,421
S16 TX (immunis* or immuniz* or vaccinat* or vaccine*) 98,421
S15 (MH "Immunisation+") 18,344
S14 S1 OR S2 OR S3 OR S4 OR S5 OR S6 OR S7 OR S8 OR S9 OR S10 OR S11 OR S12 OR S13 33,953
S13 TX hib 2,105
S12 TX "h. influenzae" 1,703
S11 TX (haemophilus influenzae OR haemophilus meningitis OR hemophilus influenzae OR hemophilus meningitis) 3,898
S10 TX (pertuss* OR whooping cough) 7,456
S9 TX (diphtheria OR dtp OR dtap OR tdap) 6,954
S8 TX tetanus 8,110
S7 TX polio* 10,862
S6 (MH "Poliomyelitis+") 2,740

S5 TX mmr 4,274
S4 TX (rubella* OR german measles) 7,151
S3 TX (epidemic parotiti* OR infectious parotiti*) 27
S2 TX mumps 5,986
S1 TX (measles OR rubeola OR rubeolla) 10,748

****

Databases: Database of Abstracts of Reviews of Effects (DARE) and Cochrane Database of Systematic Reviews (CDSR) and NHS EED
Search Date: 31 May 2016

ID Search
#1 (measles or rubeola or rubeolla)
#2 (mumps or epidemic parotiti* or infectious parotiti*)
#3 (rubella* or german measles)
#4 mmr
#5 polio*
#6 tetanus
#7 (diphtheria* or dtp or dtap or tdap)
#8 (pertuss* or whooping cough)
#9 (haemophilus meningitis or hemophilus meningitis)
#10 (haemophilus influenzae or (hemophilus influenzae))
#11 h. influenzae
#12 hib
#13 #1 or #2 or #3 or #4 or #5 or #6 or #7 or #8 or #9 or #10 or #11 or #12
#14 (immunis* or immuniz* or vaccinat* or vaccine*)
#15 #13 and #14
#16 #15 in Other Reviews
#17 #15 in Cochrane Reviews (Reviews and Protocols)
#18 #15 in Economic Evaluations

****

Databases: PubMed
Search Date: 31 May 2016

(((((((measles OR rubeola OR rubeola OR mumps OR epidemic parotiti* OR infectious parotiti* OR rubella* OR german measles OR mmr OR polio OR poliomy* OR poliovir* OR tetanus OR diphtheria* OR dtp OR dtap OR tdap OR pertuss* OR whooping cough OR haemophilus meningitis OR hemophilus meningitis OR haemophilus influenzae OR hemophilus influenza OR h. influenzae OR hib))) AND ((immunis* OR immuniz* OR vaccinat* OR vaccine*)))) AND (((CPG OR cpgs OR guidance OR guideline* OR metaanalysis OR meta-analysis OR recommend* OR review OR standard OR standards)))) AND ((publisher[sb]))) (123)

(((((((measles OR rubeola OR rubeola OR mumps OR epidemic parotiti* OR infectious parotiti* OR rubella* OR german measles OR mmr OR polio OR poliomy* OR poliovirus *or tetanus OR diphtheria* OR dtp OR dtap OR tdap OR pertuss* OR whooping cough OR haemophilus meningitis OR hemophilus meningitis OR haemophilus influenzae OR hemophilus influenza OR h. influenzae OR hib))) AND ((immunis* OR immuniz* OR vaccinat* OR vaccine*)))) AND (((cost OR costs)))) AND ((publisher[sb]))) (30)
